# Delta Hemolysin and Phenol-Soluble Modulins, but Not Alpha Hemolysin or Panton-Valentine Leukocidin, Induce Mast Cell Activation

**DOI:** 10.3389/fcimb.2016.00180

**Published:** 2016-12-12

**Authors:** Elisabeth Hodille, Charlotte Cuerq, Cédric Badiou, Françoise Bienvenu, Jean-Paul Steghens, Régine Cartier, Michèle Bes, Anne Tristan, Adriana Plesa, Vien T. M. Le, Binh A. Diep, Gérard Lina, Oana Dumitrescu

**Affiliations:** ^1^Centre International de Recherche en Infectiologie, Institut National de la Santé et de la Recherche Médicale U1111, Université Lyon 1, Centre National de la Recherche Scientifique UMR5308, Ecole Normale Supérieure de LyonLyon, France; ^2^Hospices Civils de LyonLyon, France; ^3^Laboratoire de Biochimie, Centre de Biologie SudLyon, France; ^4^Laboratoire d'Immunologie, Centre de Biologie SudLyon, France; ^5^Laboratoire de Biochimie, Groupement Hospitalier EstLyon, France; ^6^Centre National de Référence des StaphylocoquesBron, France; ^7^Laboratoire d'hématologie, Centre de Biologie SudLyon, France; ^8^Division of HIV, Infectious Diseases and Global Medicine, Department of Medicine, University of CaliforniaSan Francisco, CA, USA

**Keywords:** *Staphylococcus aureus*, mast cells, phenol-soluble modulins, delta hemolysin, virulence, accessory gene regulator, hand-transmission

## Abstract

Mast cells are located at host interfaces, such as the skin, and contribute to the first-line defense against pathogens by releasing soluble mediators, including those that induce itching and scratching behavior. Here, we show that delta-hemolysin (Hld) and phenol soluble modulins (PSMs) PSMα1 and PSMα3, but not alpha-hemolysin (Hla) or Panton-Valentine leukocidin (PVL), induce dose-dependent tryptase, and lactate dehydrogenase (LDH) release by the HMC-1 human mast cell line. Using supernatants from isogenic strains, we verified that tryptase and LDH release was Hld- and PSMα-dependent. PSMα1 and Hld production was detected in 65 and 17% of human *Staphylococcus aureus*-infected skin abscess specimens, respectively, but they were produced *in vitro* by all clinical isolates. The results suggest that Hld and PSM-α1 produced *in vivo* during *S. aureus* skin infections induce the release of mast cell mediators responsible for itching and scratching behavior, which may enhance skin to skin transmission of *S. aureus via* the hands. As Hld and PSMs are upregulated by accessory gene regulator (agr), their association may contribute to the elective transmission of *S. aureus* strains with a functional agr system.

## Introduction

Microorganisms are increasingly being recognized to have profound effects on the host they invade (Shropshire and Bordenstein, 2016). In many cases, host behaviors are altered upon infection by diverse pathogens, including bacteria (Cameron and Sperandio, 2015). These observed changes in host behavior are often thought to be beneficial to the microorganisms, as they may increase the rate of transmission (Cézilly and Perrot-Minnot, 2005).

*Staphylococcus aureus* is both a commensal and extremely versatile pathogen and one of the most common causes of bacterial community and healthcare-associated infections in humans (Lowy, 1998). *S. aureus* primarily causes skin and soft tissue infections (SSTIs), bloodstream infections, and pneumonia. The pathogenicity of *S. aureus* is determined by the extended repertoire of toxins produced by this bacteria (Kong et al., 2016). *In vitro* and *in vivo* studies in animals have identified pore-forming toxins, such as Panton-Valentine leukocidin (PVL), alpha-hemolysin (Hla), phenol-soluble modulin-alpha (PSMα), and delta-hemolysin (Hld), as major virulence factors involved in the pathophysiology of staphylococcal skin infections (Wang et al., 2007; Kobayashi et al., 2011; Lipinska et al., 2011; Syed et al., 2015). These toxins are capable of targeting a wide variety of immune cells during infection, such as human polymorphonuclear leukocytes, monocytes, and macrophages, and can significantly contribute to dampening both innate and adaptive immune response to *S. aureus* infection (Pozzi et al., 2015).

The expression of PVL, Hla, and PSMs is regulated in different manners by accessory gene regulator (agr), a quorum sensing system in *S. aureus*. Hld is encoded by RNAIII, a non-coding RNA that orchestrates the expression of most virulence factors (Novick et al., 1993), including PVL and Hla (Vandenesch et al., 1991; Dumitrescu et al., 2011). In contrast, PSMα expression is regulated by AgrA, the response regulator of the two component system that activates the agr system (Lina et al., 1998; Queck et al., 2008).

Skin to skin contact *via* the hands plays a significant role in the spread of *S. aureus* to new hosts not only in hospitals, but also in nursing homes and child care settings, among others (Bloomfield and Scott, 1997). Increasing the frequency of contact between one's hand and skin colonized or infected by *S. aureus* due to itching and scratching behavior may enhance the transmission potential of the pathogen. The induction of scratching behavior in animals during experimental skin infection by *S. aureus* was described a long time ago (Wagner et al., 1997) but only recently described in humans when skin infections induced by community-acquired methicillin-resistant *S. aureus* (CA-MRSA) were misidentified by both patients and physicians as spider bites because they were very erythematous, indurated, and itchy, sometimes with a central dermo-necrosis (Suchard, 2011).

The skin is considered a major interface of the body for the host defense, not only as a passive barrier, but also through the immune system. Innate immune cells residing in the skin, such as Langerhans cells, dendritic cells, and dermal mast cells, provide cutaneous immune surveillance (Kupper and Fuhlbrigge, 2014). Mast cells are leukocytes originating from hematopoietic progenitor cells and located at host interfaces with the environment, such as the skin, pulmonary, and digestive mucosa. Mast cell differentiation and maturation are different according to the organs in which they are located. Two major phenotypes of mature mast cells are differentiated by granule content and the receptors expressed: Phenotype T contains mainly tryptase, and phenotype TC contains mainly tryptase and chymase (Galli et al., 2011).

These cells are able to recognize pathogenic agents and trigger the inflammatory process through complex inter-cellular communication mediated by several mediators released by mast cells. Thus, mast cells participate in the first line of defense in innate immunity against pathogens, including bacteria (Abraham and St. John, 2010).

Mast cells express several receptors capable of recognizing pathogens that belong to pattern recognition receptor (PRR) family involved in the recognition of pathogen-associated molecular patterns (PAMPs), such as Toll-like receptors (TLRs) TLR1 to TLR9, Nod-like receptors (NLRs), and C-type lectin receptors (CLRs), including dectin-1 (Urb and Sheppard, 2012; St. John and Abraham, 2013). Pathogen recognition induces several activation pathways in mast cells, resulting in the release of mediators, such as intra-cytoplasmic granules (histamine, protease, tryptase, tumor necrosis factor [TNF]), lipid-derived eicosanoids (leukotrienes and prostaglandins), and cytokines or chemokines, including TNF, IL-4, and IL-6 (Abraham and St. John, 2010; Urb and Sheppard, 2012; St. John and Abraham, 2013). After bacterial invasion, mast cells preferentially release IL-8 and TNF to promote polymorphonuclear neutrophil (PMN) recruitment at the infection site (Urb and Sheppard, 2012). First, TNF, IL-6, and IL-8 promote PMN chemotaxis. Next, TNF and eicosanoid trigger an up-regulation of adhesion molecules on the endothelial cell surface and vascular permeability, allowing PMN adhesion to endothelial cells and diapedesis (Abraham and St. John, 2010). In addition, mast cells can directly kill pathogens *via* the production of antimicrobial peptides known as cathelicidins (Di Nardo et al., 2003).

Mast cells can recognize bacteria through TLR-2 and TLR-4, which recognize the lipopolysaccharides of Gram-negative bacteria and peptidoglycans of Gram-positive bacteria, and through TLR-5, which recognizes flagellin (Abraham and St. John, 2010). Mast cell mediators are also known to activate neuroreceptors on sensory nerve fibers involved in the induction of pruritus (Ständer et al., 2008).

Only a few studies have examined the interaction between *S. aureus* and mast cells. *S. aureus* induces mast cell activation through peptidoglycan-sensing by TLR2 (Feng et al., 2007). Two recent studies also confirmed that *S. aureus*-exposed mast cells release pro-inflammatory cytokines, such as IL-8, IL-3, IL-13, and TNF-α (Rönnberg et al., 2014; Swindle et al., 2015). Furthermore, *S. aureus* internalization by mast cells is enhanced by Hla and induces the release of extracellular traps, antimicrobial compounds, and pro-inflammatory cytokines (Abel et al., 2011; Goldmann et al., 2016; Johnzon et al., 2016). Nakamura et al. (2013) also demonstrated in a mouse model that *S. aureus* Hld induces skin mast cell activation, promoting the establishment of atopic dermatitis, an allergic skin disease. However, no data are yet available on the effect of other staphylococcal toxins involved in skin pathophysiology on human mast cells.

The aim of our study was to investigate whether *S. aureus* produces virulence factors during skin infection that may increase the rate of transmission by stimulating itching and scratching behavior through mast cell activation. We examined the impact of virulence factors involved in skin infection pathophysiology on skin mast cell activation and verified there *in vivo* production in human *S. aureus* skin abscess samples.

## Materials and methods

### Staphylococcal virulence factors and cells

The staphylococcal virulence factors used in this study were PVL, Hla, Hld, PSMα1, and PSMα3. The recombinant proteins LukS-PVL, LukF-PVL, and Hla were produced in our laboratory and resuspended in phosphate buffered saline (PBS) as described previously (Perret et al., 2012). Recombinant PVL activity on the plasma membranes of human polymorphonuclear leukocytes (PMNs) was verified on human PMNs before the study (Supplementary Figure 1). Briefly, purified LukS-PV and LukF-PV were diluted in RPMI to equimolar concentrations (0.05–0.5 μg/mL, i.e., 0.0015–0.015 μM) and mixed with human PMNs (10^6^ cells) in the presence of 500 ng propidium iodide (PI). The percentage of PI-positive PMNs was determined by flow cytometry (Becton Dickinson Accuri C6 Flow Cytometer) at 5, 10, 15, 20, 25, and 30 min.

The activities of alpha-hemolysin preparations were also confirmed by cell permeability assays using rabbit erythrocytes (Supplementary Figure 2). Recombinant Hla was diluted in PBS (0.001–10 μg/mL, i.e., 3 × 10^−5^–0.3 μM) and mixed at equal volume in a solution of 2% washed rabbit erythrocytes (bioMérieux). After 60 min of incubation at 37°C, samples were centrifuged at 200 × g for 5 min and the OD_405nm_ of the supernatants measured. The percentage hemolysis of each sample was compared to that of erythrocytes lysed with 1% Triton-X.

PSMα1, PSMα3, and Hld were synthesized in the N-formylated form by Genecust Europe® (Luxembourg) with a purity greater than 90% and resuspended in PBS before their use. The HMC-1 human mast cell line was kindly provided by Olivier Lortholary (Pasteur Institute, Paris, France) and cultured in Iscove's modified Eagle's medium (IMDM) supplemented with 10% fetal calf serum (FCS) and 40 μg/mL gentamicin in 5% CO_2_ at 37°C. For cell experiments, HMC-1 cell cultures were adjusted to 1 × 10^6^ cells/mL in IMDM without FCS.

### *S. aureus* strains and bacterial culture

Twenty-three *S. aureus* clinical strains isolated from SSTIs were provided by the French National Reference Center (NRC) for Staphylococci. For each strain, the agr group, MLST-clonal complex or sequence type (ST), *mecA*, Luk-PV gene, Hla, and Hld were detected using a *S. aureus* microarray genotyping kit (Alere, Jouy-en-Josas, France) as described previously (Monecke et al., 2009) (Table 1). Moreover, isogenic mutants of a *S. aureus* USA300-0114 clinical strain, SF8300, were constructed using the oligonucleotides in Supplemental Table S1 and the pKOR1 allelic replacement mutagenesis system as described previously (Bae and Schneewind, 2006; Diep et al., 2010). SF8300Δ*psm*α*1–4* and SF8300Δ*agrA* mutants contain in-frame deletions of genes encoding PSM-alpha types α1-α4 and AgrA. SF8300Hld(3G > T) mutant contains a single nucleotide change that changes the start codon ATG to ATT to block the initiation of its translation. Colonies of SF8300WT, SF8300Δ*psm*α*1–4*, and SF8300Hld(3G > T) were hemolytic on blood agar plate suggesting that agr was functional in these mutants. AgrA-knockout induces an alteration of the agr system and PSMα1–4 production (Peschel and Otto, 2013). *S. aureus* supernatants were prepared by growing bacteria adjusted to 0.5 MacFarland standard in 3 mL of CCY broth in a rotary shaker (190 rpm) at 37°C for 22 h, followed by centrifugation for 10 min at 3000 × g to obtain a pellet. Supernatants were stored at −80°C until needed.

**Table 1:** **Epidemiological characteristics of ***S. aureus*** strains isolated from SSTIs**.

***S. aureus* isolate**	**Strain characteristics**			
	**agr group**	**MLST or Clonal Complex**	***mecA* detection**	**luk-PV detection**
ST 2012 0567	1	152	−	+
ST 2014 0236	1	398	−	−
ST 2011 1376	1	398	−	−
ST 2013 0774	1	398	−	−
ST 2008 1085	1	188	−	+
ST 2012 1286	1	188	−	+
ST 2013 1093	1	22	−	+
ST 2007 1243	3	30	−	+
ST 2014 0589	1	8	−	−
ST 2014 1539	1	45	−	−
ST 2013 1442	1	97	−	−
ST 2012 0823	1	22	−	−
ST 2012 1497	1	152	−	+
ST 2014 0351	1	97	−	−
ST 2007 1031	3	80	+	+
ST 2011 1384	2	5	+	−
ST 2015 1708	1	398	−	−
ST 2012 0341	3	80	+	+
ST 2007 0901	3	30	−	+
ST 2010 1173	1	8	+	+
ST 2010 2118	3	80	+	+
0150331644	3	30	−	−
ST 2011 0395	2	5	−	−
ST 2013 1215	1	45	−	−
ST 2014 1054	1	45	−	−
ST 2015 1706	1	25	−	−
ST 2012 0150	1	45	−	−
ST 2009 1153	2	5	−	−
ST 2014 1173	3	80	+	+
ST 2012 1322	3	80	−	+
ST 2014 0441	3	30	−	−
HT 2004 1093	3	88	−	+
ST 2010 1734	2	15	−	−
ST 2014 1524	2	2482	−	+
ST 2015 1705	3	30	−	−
ST 2013 1745	1	8	−	−
ST 2014 1033	2	5	+	−
ST 2014 0409	3	30	−	+
ST 2013 1301	2	15	−	−
ST 2015 1710	3	30	−	−
ST 2011 0514	3	30	−	−
ST 2009 0867	1	8	−	−
ST 2015 1711	3	30	−	−
ST 2015 1707	2	5	−	−
ST 2014 1501	3	30	−	−
ST 2015 1709	2	5	−	−

*Underlined isolates correspond to clinical samples analyzed for toxin quantification*.

### Clinical samples

The study was conducted in accordance with the guidelines of the ethical committees of the participating hospital, Centre Hospitalier Lyon Sud (Hospices civils de Lyon, Pierre Bénite, France) or Groupement Hospitalier Est (Hospices civils de Lyon, Bron, France) (DC-201-1306). Specimens from human *S. aureus* skin abscesses were collected as part of the routine management of patients and stored at −80°C in Eppendorf tubes. A total of 23 samples were obtained and analyzed. For one patient, two samples were collected. For the 23 *S. aureus* isolates, characterization by microarray (Table 1) and culture supernatant collection were carried out as described above.

### Mast cell activation/degranulation and lysis assay

HMC-1 cells (1 × 10^6^ cells/mL) were incubated in Eppendorf tubes with PVL (0.05, 0.5, or 5 μg/mL, i.e., 0.0015, 0.015, 0.15 μM respectively), Hla (10 μg/mL, i.e., 0.3 μM), PSMα1, PSMα3, Hld (0.1, 0.5, 1, 5, 10, 25, 50, 100, or 200 μg/mL, i.e., around 0.04, 0.2, 0.4, 2, 4, 9, 20, 40, or 80 μM respectively), SF8300 wild-type (WT), SF8300Δ*psm*α*1–3*, SF8300Hld(3G > T), or SF8300Δ*agrA* supernatants (20, 10, 5, or 1% v/v) for 3 h at 37°C. Toxin concentrations were inferred from previous studies on human PMNs (Löffler et al., 2010) and preliminary results (data not published). The cells were pelleted by centrifugation at 200 × g for 10 min and the supernatants collected to measure tryptase by Immunocap (Phadia®) assay and lactate dehydrogenase (LDH by Architect c16000 (Abbott®) assay. Mast cell degranulation and lysis were evaluated through tryptase release and LDH release, respectively, and expressed as percentages using the following formula: $\%=\frac{\text{Tryptase Test}-\text{Tryptase Tneg}}{\text{Tryptase Tpos}-\text{Tryptase Tneg}}\times \;100$. The negative control (Tneg) was obtained using untriggered cell buffer and the positive control (Tpos) using cell lysis solution (Promega®).

### PVL and Hla quantification in bacterial supernatants

The PVL level in the supernatant was quantified using a specific ELISA kindly provided by bioMérieux (R&D Department, Marcy l'Étoile, France) as described elsewhere (Badiou et al., 2008). The concentration of Hla in the supernatant was quantified using a specific sandwich-type ELISA kindly provided by GlaxoSmithKline Vaccines that targets Hla with a solid-phase GlaxoSmithKline (GSK) monoclonal antibody, anti-Hla. The antibody–antigen complex was detected by a GSK polyclonal rabbit anti-Hla antibody followed by a peroxidase-conjugated goat anti-rabbit antibody as described previously (Otto et al., 2013).

### PSM quantification in bacterial supernatants and clinical specimens

Bacterial supernatants were thawed at 4°C, diluted 1:5 with methanol at 4°C, and incubated for 10 min at 4°C. After centrifugation at 10,000 × g for 5 min, the supernatants were recovered for PSM quantification. Clinical specimens were thawed to 4°C, vortexed for 1 min, sterilized by 1 h of heating at 94°C, cooled on ice to 4°C, diluted 1:5 with methanol at 4°C, and incubated for 10 min at 4°C. After centrifugation at 10,000 × g for 5 min, the supernatants were recovered for PSM quantification. PSMα1, PSMα3, and Hld were quantified by high-performance liquid chromatography mass spectrometry (HPLC-MS) in an Agilent® system using an adaptation of a method described elsewhere (Wang et al., 2007).

### Statistical analysis

*T*-tests, ANOVA, Kruskal-Wallis tests following by Bonferroni-adjusted Wilcox tests or *t*-tests, and a Pearson correlation test were performed to compare mast cell degranulation and mast cell lysis, as well as PSM and Hld production. *P* ≤ 0.05 was considered significant.

## Results

### The effect of toxins on HMC-1 cells

**Figure 1:**
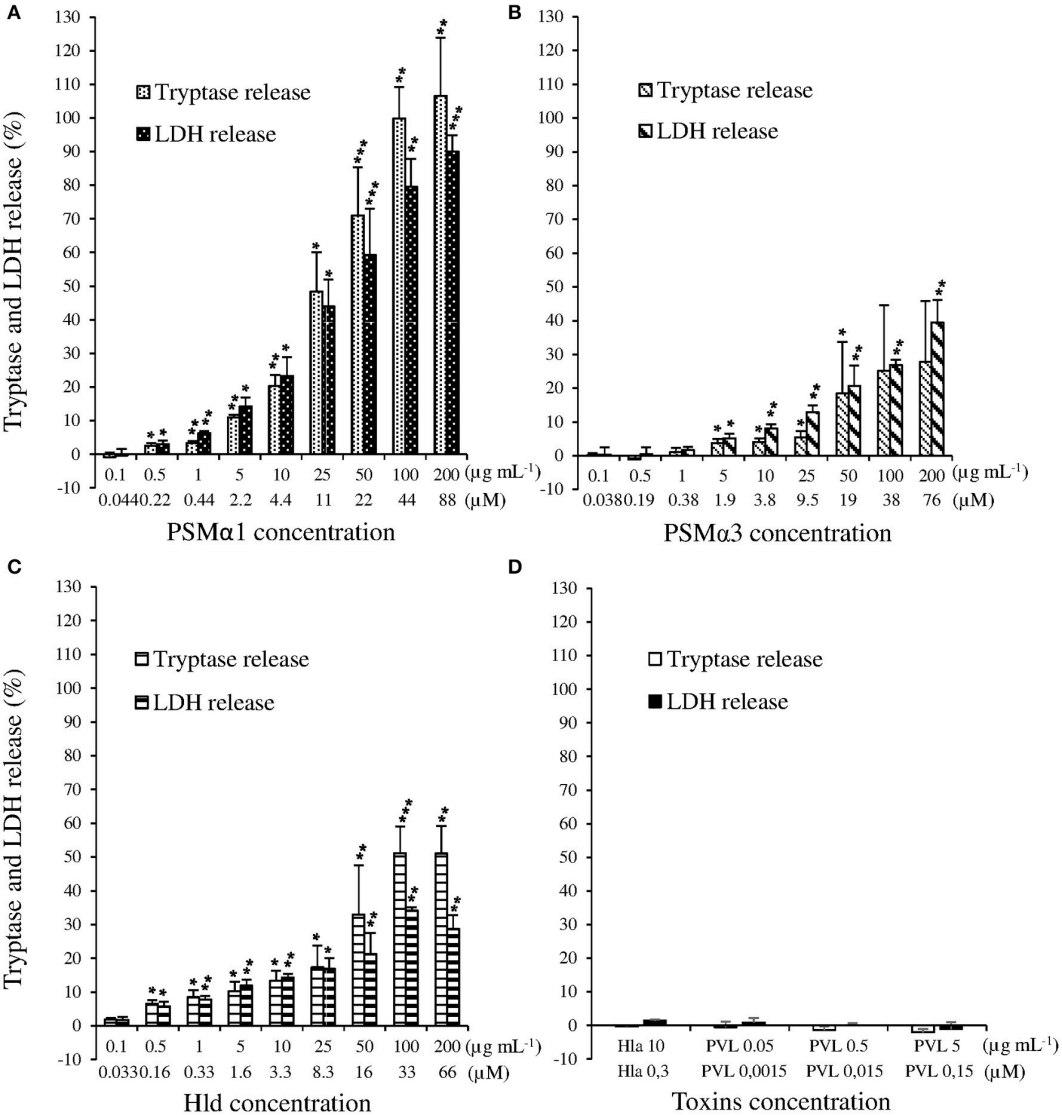
**(A)** Effect of *S. aureus* PSMα1, **(B)** PSMα3, **(C)** Hld, and **(D)** PVL and Hla on human mast cells. HMC-1 cells were incubated with increasing doses of PSMα1, PSMα3, Hld (from 0.1 μg/mL, i.e., around 4 × 10^−8^ M to 200 μg/mL, i.e., around 8 × 10^−5^ M), PVL (from 0.05 μg/mL, i.e., 1.5 × 10^−9^ M to 5 μg/mL, i.e., 1.5 × 10^−7^ M), or Hla (3 × 10^−7^ M) for 3 h at 37°C. Tryptase and LDH release were measured in cell supernatants by Immunocap (Phadia®) and Architect (Abbot®), respectively. The percentage of mast cell degranulation and mast cell lysis was calculated as $\frac{\text{Test}-\text{Tneg}}{\text{Tpos}-\text{Tneg}}\;\times \;100$. The negative control and positive control were performed with cell buffer and lysis buffer, respectively. The values represent the mean + *SD* of at least three independent experiments. To evaluate the dose-dependency on LDH and tryptase release, we performed Kruskal-Wallis tests for PSMα1, PSMα3, and Hld (*p* < 0.001 for the three toxins). To evaluate the lowest concentration inducing LDH or tryptase release that was significantly different from Tneg, *t*-tests of mean to 0 (Tneg) were performed for each toxin concentration. ^*^ *p* ≤ 0.05, ^**^ *p* ≤ 0.01, ^***^ *p* ≤ 0.001.

Because *in vitro* and *in vivo* studies in animals have been implicated PSMα1, PSMα3, Hld, Hla, and PVL in the pathophysiology of skin infections, we examined their effect on HMC-1 cells by measuring the tryptase and LDH release. The range of concentrations used for each toxin was inferred from the literature (Badiou et al., 2008; Löffler et al., 2010). We observed that PSMα1, PSMα3, and Hld induced tryptase and LDH release from HMC-1 cells in a dose-dependent manner (Kruskal-Wallis tests, *p* < 0.001 for PSMα1, PSMα3, and Hld; Figures 1A–C). PSMα1 was more active than PSMα3 and Hld. The release of both LDH and tryptase was significantly higher with PSMα1 than with PSMα3 and Hld (*p* < 0.05), with the maximal release of tryptase and LDH in the presence of 200 μg/mL toxin (i.e., 88, 76 and 66 μM for PSMα1, PSMα3, and Hld respectively), achieving levels as high as 106.58 ± 17.29 and 89.99 ± 4.89% of the positive control with PSMα1, 27.69 ± 18.09 and 39.41 ± 6.74% with PSMα3, and 51.00 ± 8.25 and 28.66 ± 4.08% with Hld. The lowest toxin concentration inducing tryptase and LDH release was 0.5 μg/mL for PSMα1 (i.e., 0.22 μM) and Hld (i.e., 0.16 μM) and 5 μg/mL for PSMα3 (i.e., 1.9 μM). Using PSMα1, PSMα3, and Hld, tryptase levels correlated with the LDH levels, suggesting that tryptase release was directly linked to mast cell lysis (Supplementary Figure 3).

In contrast to PSMs and Hld, PVL and Hla did not induce tryptase or LDH release by HMC-1 cells, even when applied at high concentrations (5 μg/mL, i.e., 0.15 μM) (Figure 1D). This negative data were not associated with the absence of activity in PVL and Hla preparations because they caused membrane disruption of humans PMNs and rabbit erythrocytes, respectively (Supplementary Figures 1, 2).

### Effect of *S. aureus* supernatants on HMC-1 cells

**Figure 2:**
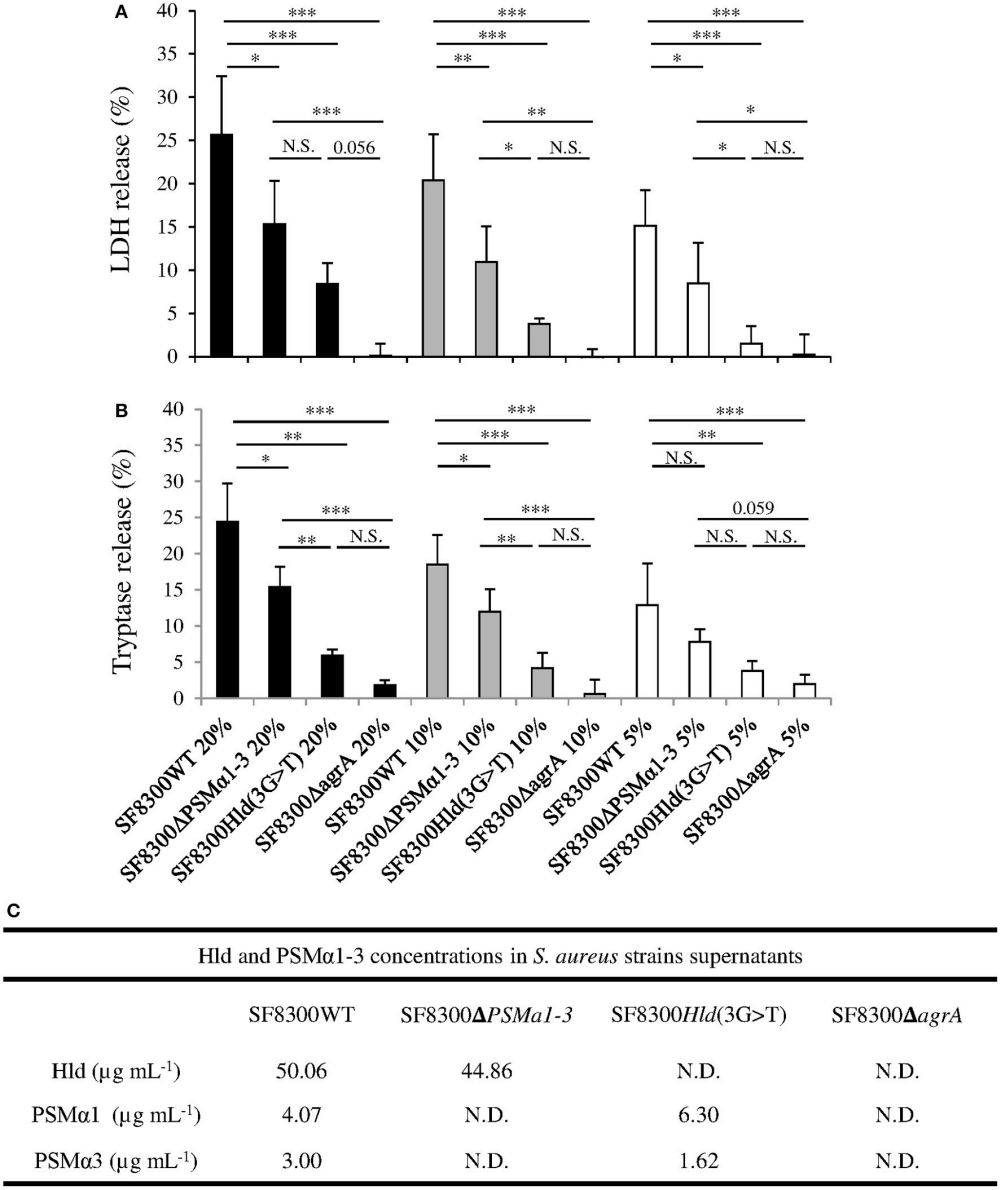
**Effect of SF8300WT, SF8300Δ***psm***α***1-3***, SF8300Hld(3G >T), and SF8300Δ***agrA*** on human mast cells**. HMC-1 cells were incubated with 20, 10, or 5% v/v *S. aureus* supernatants for 3 h at 37°C. **(A)** LDH and **(B)** tryptase release was measured in cell supernatants by Architect (Abbot®) and Immunocap (Phadia®), respectively. The percentage of mast cell lysis and mast cell degranulation was calculated as $\frac{\text{Test}-\text{Tneg}}{\text{Tpos}-\text{Tneg}}\;\times \;100$. The negative control and positive control were performed with medium and lysis buffer, respectively. The values represent the mean + SD of at least three independent experiments. **(C)** Hld, PSMα1, and PSMα3 concentrations in in each supernatant. To compare LDH and tryptase release by HMC-1 cells challenged with SF8300WT, SF8300Δ*psm*α*1-3*, SF8300Hld(3G > T), or SF8300Δ*agrA* supernatants, we performed *t*-test with Bonferroni correction followed by ANOVA. ^*^ *p* ≤ 0.05, ^**^ *p* ≤ 0.01, ^***^ *p* ≤ 0.001. N.S., not significant; N.D., not detected (toxin concentrations were less than the detection limit of 0.1 μg/mL).

We quantified Hla, PVL, PSMα1, PSMα3, and Hld in supernatants of SF8300 and its isogenic mutants to confirm their toxin production profiles. As expected, the concentrations of Hla and PVL in SF8300WT, SF8300Hld(3G > T), and SF8300Δ*psm*α*1–4* supernatants were similar (Hla: 0.4, 0.4, 0.4 μg/mL; PVL: 18, 25, 26 μg/mL), whereas only a small amount of Hla and PVL was detected in SF8300Δ*agrA* supernatant (0.001 and 0.2 μg/mL, respectively). SF8300WT produced PSMα1 (4.07 ± 0.41 μg/mL), PSMα3 (3.00 ± 0.10 μg/mL), and Hld (50.06 ± 6.45 μg/mL), whereas SF8300Hld(3G > T) produced only PSMα1 (6.30 ± 0.64 μg/mL) and PSMα3 (1.62 ± 0.05 μg/mL), SF8300Δ*psm*α*1–4* produced only Hld (44.86 ± 5.70 μg/mL), and SF8300Δ*agrA* produced none of the mediators (Figure 2).

When we examined the effect of the supernatant from the *S. aureus* SF3800 strain on HMC-1 cells, we observed that LDH and tryptase concentratons were consistent with the effect of synthetic peptides PSMα1, PSMα3, and Hld. LDH and tryptase release by HMC-1 cells was concentration-dependent (from 20 to 5% v/v; ANOVA, *p* < 0.01) and varied between SF3800WT and its isogenic mutants (Figure 2). The staphylococcal supernatant-induced levels of LDH and tryptase release (using 20 to 5% v/v) in descending order were from SF8300WT, SF8300Δ*psm*α*1–4*, SF8300Hld(3G > T), and SF8300Δ*agrA*. Taken together, these results indicate that LDH and tryptase release by HMC-1 cells challenged with *S. aureus* supernatants was mainly associated with Hld production and secondary to PSMα1 and PSMα3 production.

### PSMα and Hld quantification in clinical specimens

As only PSMα1, PSMα3, and Hld induced tryptase and LDH release by HMC-1 cells, we explored their production during human skin infection (Table 2). Hld was detected in 15 of the 23 pus samples at concentrations ranging from 0.1 to 19.6 μg/mL (median 0.85 μg/mL). PSMα1 was detected in only four of the pus samples at concentrations ranging from 0.1 to 3.9 μg/mL (median 0.31 μg/mL). Higher concentrations of Hld and PSMα1 were detected in the same pus samples. Twelve of the 15 (80%) Hld-positive specimens reached toxic concentrations for HMC-1 cells (i.e., 0.5 μg/mL), but only one pus sample contained a toxic concentration of PSMα1 for HMC-1 cells. Finally, PSMα3 was detected in none of the tested clinical specimens.

**Table 2:** **Hld, PSMα1, and PSMα3 concentrations in human skin abscesses and bacterial supernatants**.

***S. aureus* isolate**	**Pus (μg mL^−1^)**			**Supernatant (μg mL^−1^)**		
	**Hld**	**PSMα1**	**PSMα3**	**Hld**	**PSMα1**	**PSMα3**
ST 2014 0441	19.57	3.92	ND	68.84 ± 9.19	14.45 ± 4.28	0.11 ± 0.02
ST 2012 0567	3.69	ND	ND	199.47 ± 67.67	33.73 ± 8.48	14.02 ± 4.96
ST 2014 1173	1.33	ND	ND	77.53 ± 0.05	5.76 ± 2.11	2.89 ± 0.71
ST 2013 0774	1.31	ND	ND	171.55 ± 14.38	12.16 ± 2.70	6.07 ± 2.67
ST 2015 1706	1.26	0.40	ND	88.49 ± 12.44	8.45 ± 1.72	2.94 ± 0.82
ST 2013 1442	1.13	ND	ND	141.12 ± 13.58	28.52 ± 2.52	17.10 ± 7.06
ST 2012 1497	0.90	ND	ND	128.72	8.91	2.07
ST 2015 1705	0.85	ND	ND	52.50 ± 20.31	1.12 ± 0.89	0.07 ± 0.01
015033164401	0.82	ND	ND	98.75 ± 0.69	22.04 ± 0.32	0.08 ± 0.01
ST 2014 0409	0.67	ND	ND	37.79 ± 4.29	0.37 ± 0.11	0.06 ± 0.03
ST 2014 0351	0.59	0.22	ND	123.00 ± 11.62	10.42 ± 0.71	2.65 ± 0.06
ST 2015 1711	0.52	ND	ND	22.15 ± 9.55	0.33 ± 0.08	0.07 ± 0.01
ST 2014 1033	0.31	0.19	ND	43.56 ± 2.02	9.02 ± 4.62	2.58 ± 1.58
ST 2012 1322	0.26	ND	ND	71.81	1.61	0.42
ST 2014 0589	0.12	ND	ND	143.72 ± 20.12	10.95 ± 2.94	4.13 ± 0.21
ST 2014 1539	ND	ND	ND	143.53 ± 25.19	20.55 ± 11.23	6.70 ± 3.86
ST 2015 1708	ND	ND	ND	116.15 ± 21.28	7.95 ± 2.25	4.80 ± 1.66
ST 2014 1054	ND	ND	ND	92.21 ± 4.28	10.19 ± 1.27	2.92 ± 0.46
ST 2014 1524	ND	ND	ND	53.99 ± 23.55	2.20 ± 1.53	0.44 ± 0.20
ST 2015 1710	ND	ND	ND	33.09 ± 2.19	4.43 ± 0.19	0.08 ± 0.01
ST 2015 1707	ND	ND	ND	14.70 ± 2.94	0.47 ± 0.17	0.15 ± 0.04
ST 2014 1501	ND	ND	ND	7.66 ± 4.91	0.14 ± 0.04	0.07 ± 0.03
ST 2015 1709	ND	ND	ND	4.38 ± 3.05	<0.1	0.07 ± 0.03

*ND, not detected*.

### *In vitro* PSMα and Hld production by *S. aureus* strains

Forty-six *S. aureus* clinical isolates from SSTIs were studied for PSMα1, PSMα3, and Hld production, including 23 from the clinical specimens analyzed for *in vivo* PSM and Hld production. The other 23 isolates were from the French staphylococci NRC collection with no pus available. Strains belonged to different agr groups and clonal complexes (Table 1): 45.6% (21/46) *S. aureus* strains in agr group 1, 19.6% (9/46) in agr group 2, and 34.8% (16/46) in agr group 3. A majority (39/46) of strains were methicillin-susceptible *S. aureus* (MSSA), and five of seven methicillin-resistant *S. aureus* (MRSA) strains were *luk-PV*-positive. Four of the latter were community-acquired MRSA strains belonging to the European ST 80 clone, and one belonged to the ST8 USA300 clone. The remaining *luk-PV*-positive *S. aureus* strains (*n* = 11) were MSSA.

**Figure 3:**
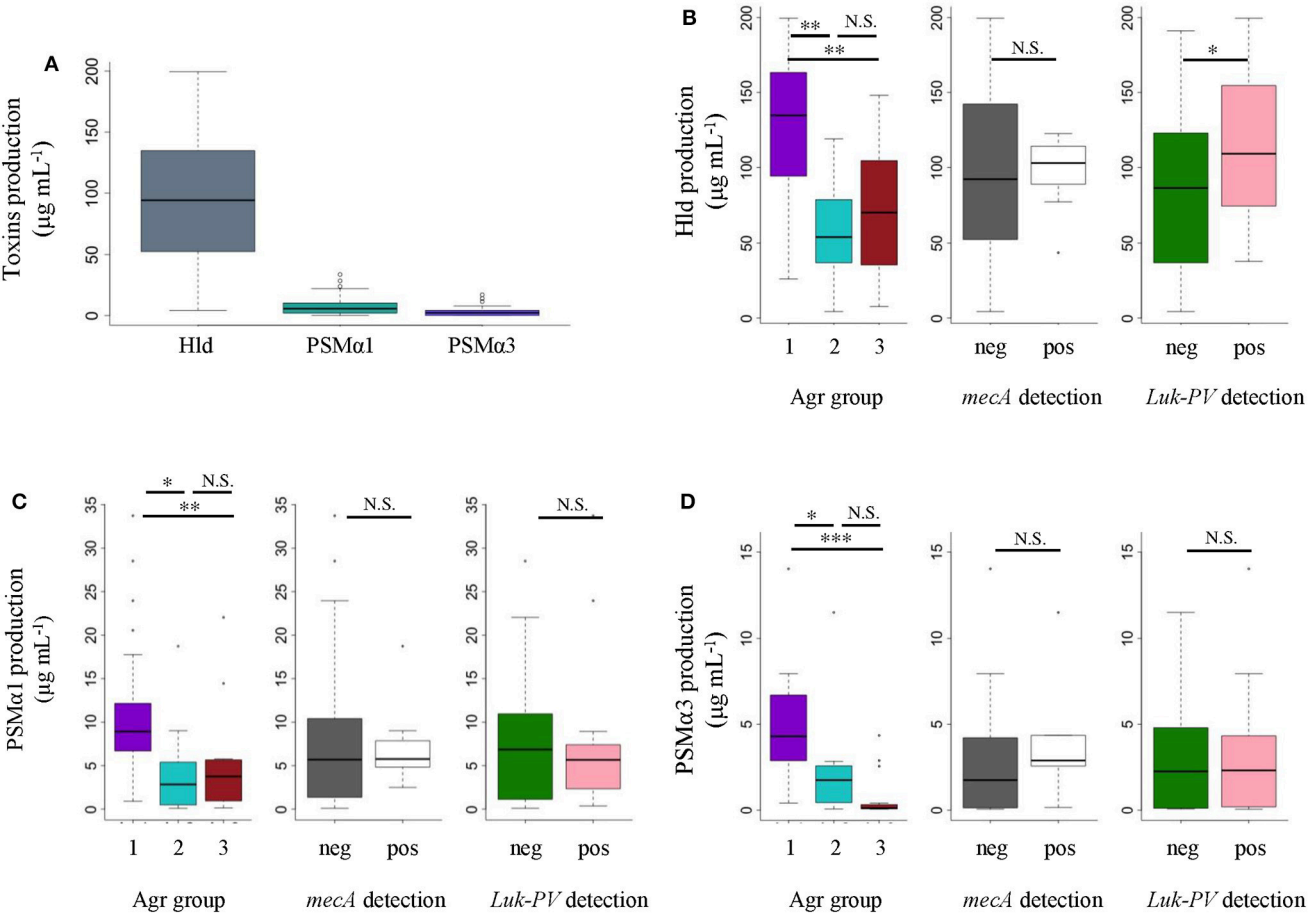
**Comparisons of ***in vitro*** PSMα1, PSMα3, and Hld production in 46 ***S. aureus*** SSTI strains**. HPLC-MS was performed on supernatants from 22-h cultures. We performed *t*-tests Bonferroni correction followed by ANOVA, and *t*-tests when it was possible (parametric) or Kruskal-Wallis tests followed by Wilcox tests with Bonferroni correction (non-parametric). **(A)** Comparison of toxin production. **(B)** Comparison of *in vitro* Hld, **(C)** PSMα1, and **(D)** PSMα3 production according to agr group, methicillin resistance, and *luk-PV* carrier. ^*^ *p* ≤ 0.05, ^**^ *p* ≤ 0.01, ^***^ *p* ≤ 0.001. N.S., not significant.

All *S. aureus* strains expressed PSMα1, PSMα3, and Hld *in vitro*, though at different levels (Figure 3). Nevertheless, the mean Hld production (94.51 ± 52.02 μg/mL) was much higher than the mean PSMα1 production (8.10 ± 7.91 μg/mL), which was higher than the mean PSMα3 production (3.09 ± 3.79 μg/mL) (Figure 3A). The *in vitro* production levels of the three toxins were not different between MRSA and MSSA (Figures 3B–D). We did not notice any difference in the *in vitro* PSMα1 and PSMα3 production levels between *luk-PV*-positive (*n* = 16) and *luk-PV*-negative (*n* = 30) *S. aureus* strains (Figures 3C,D). However, *luk-PV*-positive strains produced more Hld (113.05 ± 46.42 μg/mL) than *luk-PV*-negative strains (84.62 ± 52.86 μg/mL, *p* < 0.05; Figure 3B). We also observed that *S. aureus* from agr group 1 produced higher levels of Hld, PSMα1, and PSMα3 than *S. aureus* from agr groups 2 and 3 (*p* < 0.05; Figures 3B–D). No significant difference was found in PSMα1, PSMα3, and Hld production between community-acquired MRSA and the other *S. aureus* strains.

Regarding the 23 *S. aureus* strains isolated from pus, we found higher toxin concentrations in culture supernatants than clinical specimens (Table 2). *In vitro* toxin production ranged from 4.38 to 199.47 μg/mL (median 77.53 μg/mL) for Hld, from <0.1 to 33.73 μg/mL (median 8.2 μg/mL) for PSMα1, and from 0.06 to 17.1 μg/mL (median 2.07 μg/mL) for PSMα3. No correlation was observed between *in vivo* and *in vitro* Hld quantification (Supplementary Figure 4).

## Discussion

Despite progress in our knowledge of *S. aureus* pathophysiology, virulence factors that favor its transmission remain enigmatic. Hands play a significant role in the spread of *S. aureus* to new hosts through contact with skin colonized or infected by *S. aureus*. In the skin, dermal mast cells act in close interaction with nerve fibers and contain substances that act as mediators of itching (Ständer et al., 2008). In this study, we examined whether *S. aureus* pore-forming toxins that are involved in the pathophysiology of skin infection can induce mast cell activation/lysis. We used HMC-1 cells derived from a patient with leukemia. Even though this cell line has two major deficiencies (cytokine-independence and lack of a functional FcεR), it is widely used and a faithful *in vitro* model of normal mast cells (Drexler and MacLeod, 2003). We verified that HMC-1 cells produce tryptase, one of the best indicators of mast cell activation (Schwartz et al., 2003; Guhl et al., 2010), and express CD88 similar to human skin mast cells (Füreder et al., 1995; Werfel et al., 1996) (Supplementary Figure 5).

Our results clearly demonstrate that PSMα1, PSMα3, and Hld induce dose-dependent tryptase and LDH release by HMC-1 cells. We suspect that tryptase liberation was mainly the result of mast cell lysis. In contrast, PVL and Hla did not induce human HMC-1 cell degranulation or lysis. This finding was unexpected because König et al. (1995) reported that PVL induces histamine release by human basophilic granulocytes, immune cells that are morphologically and functionally related to mast cells, especially given that the HMC-1 cell line expresses C5aR, the PVL receptor (König et al., 1995; Spaan et al., 2013). Two major hypotheses are possible to explain this discrepancy. Either HMC-1 cells do not express enough CD88 to make the cell sensitive to PVL and/or there is an additional specific co-receptor necessary for PVL to be active that is missing from HMC-1 cells. Supplementary investigations should be performed to test these hypotheses.

We found a similar cytotoxic effect of PSMα1 and PSMα3 on HMC-1 cells as those previously reported with human neutrophils. The lethal concentration 50% was achieved in both mast cells and human neutrophils at 25 μg/mL PSMα1 (Löffler et al., 2010). However, for PSMα3, we observed only 20% lysis of mast cells at the concentration that results in the 80% lysis of neutrophils (i.e., 50 μg/mL) (Löffler et al., 2010). We observed the same level of Hld activity against mast cells as Nakamura et al.; using 10 μg/mL we measured 13.30 ± 3.01% tryptase release, whereas they reported β-hexosaminidase release of ~10% (Nakamura et al., 2013). The only difference was that β-hexosaminidase release by Hld seems to not be related to cell lysis of the mouse MC/9 mast cell line, which is in contrast to what we observed with HMC-1 cells. This may be due to the different origins of the mast cell lines.

In order to determine whether PSMα1, PSMα3, and Hld are expressed *in vivo*, 23 pus samples recovered from human *S. aureus* skin abscesses were analyzed. We detected the presence of Hld in 65% of the analyzed clinical specimens and PSMα1 in 17% of the samples, but no PSMα3. The concentration of Hld and PSMα1 detected in pus samples was 50- and 10-fold lower, respectively, than the concentrations produced *in vitro* and did not correlate. The weakest toxin concentration *in vivo* may indicate that the toxins were degraded more rapidly in pus than in supernatants, despite similar storage procedures. Only 50% of pus samples contained sufficient Hld levels to induce HMC-1 cell lysis, but it was 100% with the *in vitro* supernatants. *In vitro* Hld production was 10-fold higher than mean PSMα1 production and 30-fold higher than PSMα3 production as described previously (Berlon et al., 2015).

Two studies recently demonstrated that PSM and Hld production by MRSA and MSSA is higher among SSTI isolates than either infectious endocarditis or hospital-acquired pneumonia (Berlon et al., 2015; Qi et al., 2016). These observations suggested that the level of PSM and Hld correlates more with the disease than the methicillin-resistant status. As PSM and Hld production is regulated by the agr, the level of PSM and Hld production may reflect agr activity and its requirement for the development of skin infection in humans, as in animal models of *S. aureus* skin infection (Montgomery et al., 2010; Cheung et al., 2011). Looking at the impact of agr alleles in our sample, strains in agr group 1 produced more Hld, PSMα1, and PSMα3 than those from groups 2 and 3, despite CA-MRSA belonging mainly to agr group 3 (Tristan et al., 2007).

The involvement of PSMα1–3 in SSTI pathogenesis was demonstrated by Wang et al., who showed that *S. aureus* strains producing PSMα are more virulent, notably through the capacity of PSMα to lyse neutrophils (Wang et al., 2007). Recently, Syed et al. highlighted the involvement of *S. aureus* PSMα, including Hld, in the induction of skin inflammation through pro-inflammatory cytokine release from keratinocytes (Syed et al., 2015). Skin mast cells are very important immune cells and participate in the fight against pathogens, but they can be deleterious when over-stimulated (Abraham and St. John, 2010; Urb and Sheppard, 2012; St. John and Abraham, 2013). This deleterious effect has been shown in mice, with Hld participating in the development of atopic dermatitis (Nakamura et al., 2013). Therefore, our results suggest that *S. aureus* produces Hld during acute skin infection, and to a lesser extent PSMα1, which induces mast cell degranulation and/or lysis directly or indirectly through skin inflammation. This human skin mast cell degranulation activates neuroreceptors on sensory nerve fibers involved in the induction of pruritus and explain the itchy feeling during *S. aureus* skin infections (Zhu et al., 2015). We speculate that the scratching behavior induced by *S. aureus* though Hld and PSM production promotes *S. aureus* transmission *via* hand-to-skin contact in the presence of *S. aureus*-infected skin lesions, thereby promoting *S. aureus* epidemicity. As highlighted previously, agr is a key regulatory system for the maintenance of *S. aureus* virulence, which is considered a key factor for host-to-host transmission (Massey et al., 2006). Moreover, Hld is encoded by RNAIII, the effector of agr (Novick et al., 1993). We hypothesize that the Hld–agr association contributes to the elective transmission of virulent *S. aureus* strains with a functional agr system. Altogether could participate in the epidemic success of certain *S. aureus* strains, such as the pandemic CA-MRSA USA300 clone that strongly expresses toxins with the help of the agr system, induces itchy skin infections, and is pandemic in the USA (DeLeo et al., 2010; Cheung et al., 2011; Suchard, 2011).

In conclusion, our results demonstrate that Hld and PSMα1 are produced by *S. aureus in vivo* and induce human mast cell degranulation and lysis. As mast cell mediators are known to be involved in the induction of pruritus, we hypothesize that *S. aureus* may promote its transmission *via* the hands through scratching of infected skin lesions prompted by Hld and PSM production.

## Ethics statement

This study was conducted according to the principles of the Declaration of Helsinki and its subsequent amendments, the guidelines for Good Clinical Practices (CPMP/ICH/135/95), the French regulations and the guidelines of the ethical committees of the participating hospital, Centre Hospitalier Lyon Sud (Hospices civils de Lyon, Pierre Benite, France), and Groupement Hospitalier Est (Hospices civils de Lyon, Bron, France) (DC-201-1306). Only specimens from human *S. aureus* skin abscesses were collected as part of the routine management of patients was examined.

## Author contributions

EH, VL, BD, MB, AT, GL, and OD designed the experiments. EH, CC, CB, FB, JS, RC, and AP performed the experiments. EH, VL, BD, GL, and OD wrote the manuscript.

## Funding

This work was supported in part by the LABEX ECOFECT (ANR-11-LABX-0048) of Université de Lyon, within the program “Investissements d'Avenir” (ANR-11-IDEX-0007) operated by the French National Research Agency (ANR).

### Conflict of interest statement

The authors declare that the research was conducted in the absence of any commercial or financial relationships that could be construed as a potential conflict of interest.
