# Supplementary material for: Delta Hemolysin and Phenol-Soluble Modulins, but Not Alpha Hemolysin or Panton-Valentine Leukocidin, Induce Mast Cell Activation
Source: Front Cell Infect Microbiol. 2016 Dec 12;6:180. doi: 10.3389/fcimb.2016.00180 (PMC5149515; doi:10.3389/fcimb.2016.00180)
Supplement: Supplementary file 1 [file DataSheet1.docx]

Supplementary Material

# Delta Hemolysin and Phenol-soluble Modulins, but not Alpha Hemolysin or Panton-Valentine Leukocidin, Induce Mast Cell Activation

Elisabeth Hodille^a,b^, Charlotte Cuerq^b,d^, Cédric Badiou^a^, Françoise Bienvenu^b,e^, Jean-Paul Steghens^b,d^, Régine Cartier^b,f^, Michèle Bes^a,b,c^, Anne Tristan^a,b,c^, Adriana Plesa^b,g^, Vien T.M. Le^h^, Binh An Diep^h^, Gérard Lina^a,b,c,^*, Oana Dumitrescu^a,b,c^

^a^ Centre International de Recherche en Infectiologie, Inserm U1111, Université Lyon 1, CNRS UMR5308, ENS Lyon, Lyon, France

^b^ Hospices Civils de Lyon, Lyon, France

^c^ Centre National de Référence des Staphylocoques, Bron, France

^d^ Laboratoire de Biochimie, Centre de Biologie Sud, Lyon, France

^e^ Laboratoire d’Immunologie, Centre de Biologie Sud, Lyon, France

^f^ Laboratoire de Biochimie, Groupement Hospitalier Est, Lyon, France

^g^ Laboratoire d’hématologie, Centre de Biologie Sud, Lyon, France

^h^ Division of Infectious Diseases and Vaccinology, School of Public Health, University of California Berkeley, California, USA

*** Correspondence:** Corresponding Author: gerard.lina@univ-lyon1.fr

# Supplementary Data

Supplementary Material

Flow cytometric analysis of CD88 expression on HMC-1

The CD88 expression on HMC-1 was analyzed by multi-parameter cytometry, using FACSCanto II flow cytometer and monoclonal antibodies (MoAb) anti-CD88 FITC (clone P12/1, AbDSerotec). HMC-1 cells (5 × 10^5^ cells in 100 µL) were incubated with 5 µL of monoclonal antibodies anti-CD88 or without (control) during 15 min at dark, then HMC-1 cells were washed with PBS buffer et pelleted at 2000×*g* for 5 min. Supernatant was removed and pellet was resuspended with 200 µL of PBS buffer and analyzed.

# Supplementary Figures and Tables

## Supplementary Figures


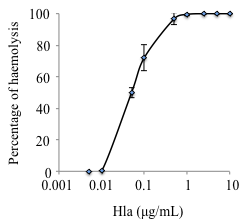


**Supplementary Figure 1.** Hla induces pore formation in rabbit erythrocytes.

0.001 µg/mL = 3x10^-11^ M; 0.01 µg/mL = 3x10^-10^ M; 0.1 µg/mL = 3x10^-9^ M ; 1 µg/mL = 3x10^-8^ M; 1 µg/mL = 3x10^-7^ M


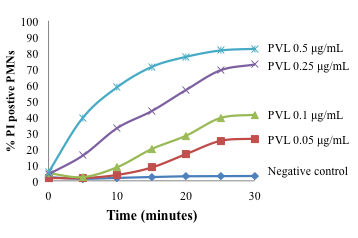


**Supplementary Figure 2.** PVL induces pore formation in human PMNs.

0.05 µg/mL = 1.5x10^-9^ M; 0.1 µg/mL = 3x10^-9^ M; 0.25 µg/mL = 7.8 x10^-9^ M;

0.5 µg/mL = 1.5X10^-8^ M

**
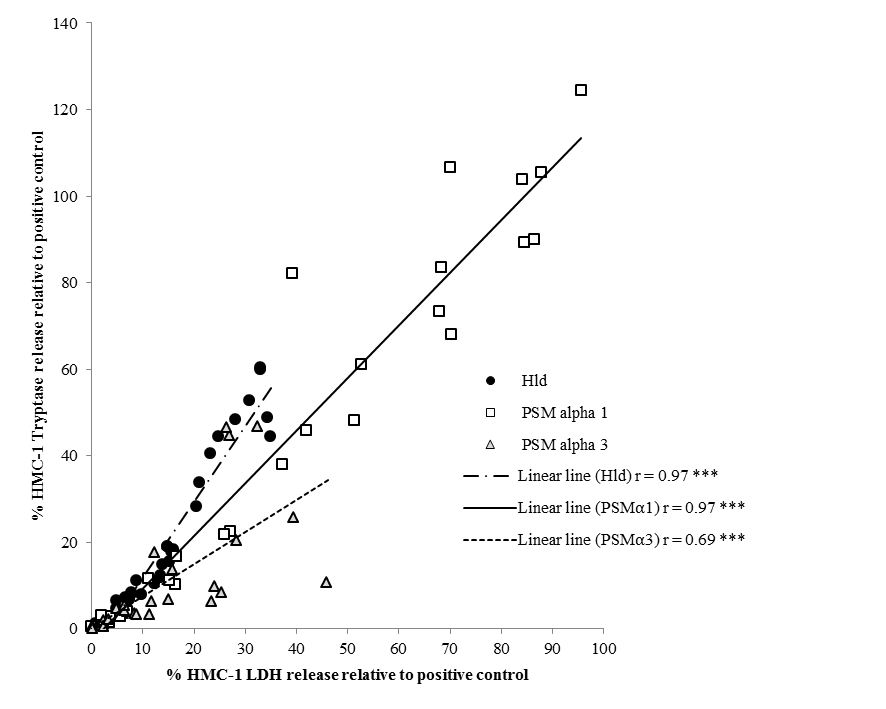
**

**Supplementary Figure 3.** Tryptase release according to LDH release for PSMα1, PSMα3, and Hld. To evaluate correlation between tryptase and LDH release, we performed correlation tests with Pearson method for each toxin. r = correlation coefficient. * p ≤ 0.05, ** p ≤ 0.01, *** p ≤ 0.001.


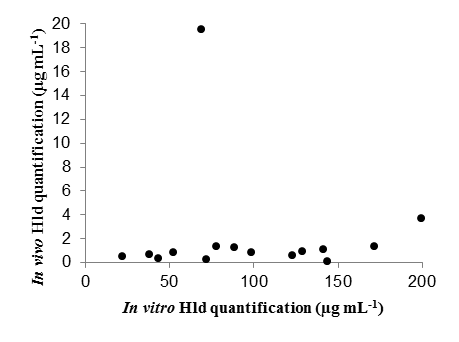


**Supplementary Figure 4.** *In vivo* Hld quantification according to *in vitro* Hld production. Hld was quantified by HPLC-MS. To evaluate correlation between tryptase and LDH release, we performed correlation tests with Pearson method for each toxin.


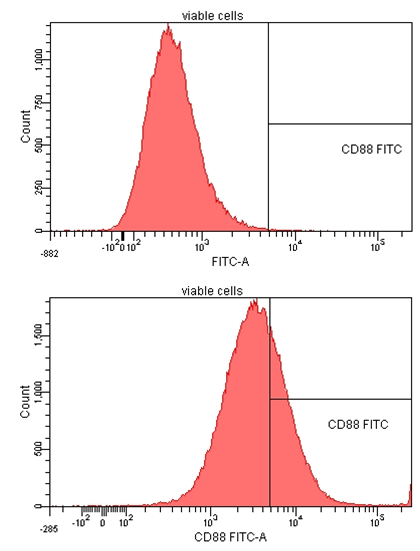


**Supplementary Figure 5.** Binding of anti-CD88 MoAb 12/1 to HMC-1 cells. FACS histograms performed on 5 × 10^5^ cells. Upper: Negative control without MoAb. Lower: anti-CD88 MoAb assay.

**Table S1.** Oligonucleotides used for constructing in-frame gene deletions using the pKOR1 allelic replacement system (Diep et al., 2010; Bae and Schnewind, 2006).

| **Oligo ID** | **Nucleotide sequence 5’ to 3’** |
| --- | --- |
| ***For inactivation of hld gene* by changing its start codon, ATG, to ATT (the G to T change is underlined)** | |
| hld-X1 | ctgtacttaataccactaattatagctg |
| hld-X2 | CACCGATTGTTGAAATGATATCTTGTGCAATTGAAATCACTCCTTCC |
| hld-X3 | GGAAGGAGTGATTTCAATTGCACAAGATATCATTTCAACAATCGGTG |
| hld-X4 | CCCAACTTATCTGTTATGATTTACG |
| hld-X5 | GGGG ACAAGTTTGTACAAAAAAGCAGGCT ccacctactatcacactctc |
| hld-X6 | GGGG ACCACTTTGTACAAGAAAGCTGGGT GATATCCTGCTCGTAGTGGTGC |
| hld-S1 | ccaaaaagaagaaggtgcatgtgc |
| hld-S2 | CATTACAAAAAAGGCCGCGAG |
| ***For in-frame deletion of genes encoding phenol soluble modulin α-type 1,2,3,4 (psmα1-4)*** | |
| psma-X1 | TACCCGCCACTCGCCAGTC |
| psma-X2 | GTATGTCCCACTAAGAACGTCCTATACCCTCCTTTGCTTATGAGTTAACTTC |
| psma-X3 | GAAGTTAACTCATAAGCAAAGGAGGGTATAGGACGTTCTTAGTGGGACATAC |
| psma-X4 | GAGCGAGTCAGCAGGATGG |
| psma-X5 | GGGG ACAAGTTTGTACAAAAAAGCAGGCT CAACCACATAAAAATGTCATGCTTG |
| psma-X6 | GGGG ACCACTTTGTACAAGAAAGCTGGGT CATCGCATACACCTGAAACAGAAG |
| psma-S1 | CAATAGTAGATTCTGTACATAATGG |
| psma-S2 | CATATCAATGGCAAATTAGACCAGC |
| ***For in-frame deletion of agrA*** | |
| agrA-X1 | gcacaccatatctaatactaaacaaagg |
| agrA-X2 | CACCGATGCATAGCAGTGTTCCTCATAAGGATTATCAGTTGCGAG |
| agrA-X3 | CTCGCAACTGATAATCCTTATGAGGAACACTGCTATGCATCGGTG |
| agrA-X4 | gaagcaaacactgcgttagc |
| agrA-X5 | GGGG ACAAGTTTGTACAAAAAAGCAGGCT gttatttcgactatcttactgcttac |
| agrA-X6 | GGGG ACCACTTTGTACAAGAAAGCTGGGT cctctgctgatatgttatttgaacc |
| agrA-S1 | gattagtatcgaaatacccgatgaag |
| agrA-S2 | gcaggattttagcaaccgatg |
